# The challenge of individualised risk assessment and therapy planning in elderly high-risk myelodysplastic syndromes (MDS) patients

**DOI:** 10.1007/s00277-012-1472-8

**Published:** 2012-05-01

**Authors:** Reinhard Stauder

**Affiliations:** Department of Internal Medicine V (Haematology and Oncology), Innsbruck Medical University, Anichstraße 35, 6020 Innsbruck, Austria

**Keywords:** Myelodysplastic syndromes (MDS), Elderly, Geriatric, Therapy, Assessment

## Abstract

Myelodysplastic syndromes (MDS) represent one of the most frequent and serious haematologic diseases of the elderly. Effective therapies exist ranging from best supportive care to haematologic stem cell transplantation (HSCT). Decision making, however, is rather complex in this group of patients because ageing is a multidimensional process involving not only physiological changes but also changes in functional, social, emotional and cognitive capacities. All these factors can have a significant impact on the efficacy and tolerability of a potential therapy and therefore have to be thoroughly assessed before deciding on individual treatment regimens. Risk assessment tools are available both to classify the stage and prognosis of MDS and to meet the needs of elderly patients. A tool explicitly focussing on elderly MDS patients, however, is still missing. The current report approached this issue by combining the well established MDS-risk score ‘International Prognostic Scoring System’ (IPSS) with the ‘Multidimensional Geriatric Assessment’ (MGA). As decision making is most complex in high-risk MDS patients, the new algorithm is presented exemplarily for this group of patients. In a first step, MDS-related risk is identified using IPSS, in a second step, patients are assigned to one of three risk categories of the MGA (go-go/fit, slow-go/vulnerable, no-go/frail). While go-go patients might be subjected to therapies comparable to those given to younger patients, in no-go patients, a palliative therapy combined with best supportive care will probably be most appropriate. In slow-go patients, age-related life expectancy taken from public age statistics should be compared to the MDS-related life expectancy. Based on this combined assessment procedure and also on treatment tolerance in terms of the expectations/wishes of the patient and his/her family, an individualised therapeutic approach should be developed. Specific treatment recommendations for these three groups of patients are given, including HSCT, azanucleosides and best supportive care. To illustrate its practicability, i.e. the implementation of the novel algorithm in clinical practice, the case of an elderly high-risk MDS patient is presented and discussed in detail. This new algorithm will facilitate the identification of the very particular needs and conditions of elderly MDS patients in clinical practice. Based on this, individually tailored therapeutic approaches can be developed—the prerequisite for the best possible clinical outcome.

## Background

The term myelodysplastic syndromes (MDSs) covers a broad spectrum of disorders, all of which result from functional abnormalities in various haematologic cell lineages that eventually lead to cytopenias. Accordingly, symptoms vary from anaemia and thrombocytopenia to neutropenia, which usually are accompanied by fatigue, a reduced overall performance status and recurrent infections. With disease progression, this chronic disorder can transform into an acute myeloid leukaemia (AML) [1, 2]. While MDS is actually a rare disease (3 to 5 cases/100,000 general population per year), it becomes rather common with increasing age (>70 years, >30 cases/100,000 per year); median age at first diagnosis is approximately 75 years. In fact, MDS represents the most frequent haematologic disease in elderly patients. In addition, the poor prognosis associated particularly with higher-risk MDS patients [International Prognostic Scoring System (IPSS) intermediate-2 and high-risk; median overall survival, 1.2 and 0.4 years, respectively] and the fact that progression to AML occurs much earlier in elderly patients than in younger ones make this disorder one of the most serious and challenging neoplastic diseases in this group of patients [1, 2].

Not only with MDS but also with any other malignancy or serious disease, optimal treatment of elderly patients can only be ensured when the high diversity of this population with respect to health status and also individual patient-related factors (see below) is recognised [3–5]. Definitions of age groups [old (>70), oldest (>85)] have been adopted to identify patients that need special attention and care [3]. Ageing, however, is a multidimensional and individual process involving not only physiological and medical but also social, emotional and cognitive changes that may hardly be reflected in chronological age [5, 6]. Therefore, health care providers should rather determine the patient’s biological age taking into account all these factors instead of chronological age when deciding on a specific treatment.

To evaluate the biological age, a geriatric assessment is required to cover all these different aspects of ageing. The so-called multidimensional geriatric assessment (MGA) approaches this issue by considering the following factors: age-adjusted life expectancy, functional reserves, comorbidities, cognitive function, social support, emotional status/depression, nutrition and polypharmacy [5]. Regarded as the most sensitive assessment parameters of function in older individuals, the ability to carry out activities of daily living (ADLs) and instrumental ADLs (IADLs) are taken into account by the MGA [7, 8]. By definition, ADLs are basic self-care activities, while IADLs are skills required for an independent life, such as housekeeping, or the ability to take medications, etc. [7, 8]. Besides these, comorbidities [9, 10] and a number of conditions termed geriatric syndromes (e.g. frequent falls) are suggested to be determined when assessing the outcome and tolerance of an elderly patient for a certain therapy [11]. Results of the MGA allow health care providers to distinguish between biologically younger patients (low risk of toxicity) that will also tolerate therapies comparable to those given to younger adults, and biologically older patients (high-risk of toxicity) that have to be treated more cautiously, e.g. with different therapeutic regimens, or by reducing the dose of a drug.

In support of this concept, a predictive score for chemotherapy toxicity, which is based on geriatric assessment variables including IADLs, falls, or social activities, was developed recently [12]. Similarly, Extermann et al. [13] developed a score that predicts haematologic and non-haematologic toxicities based on several predictors, including classical parameters of geriatric assessment like IADLs, cognition or nutritional assessment. The validity of these predictive scores in the chemotherapy treatment of haematologic malignancies has to be proven.

## Challenges in the treatment of elderly cancer patients

Despite the availability of this sensitive assessment tool, decision making in the management of elderly cancer patients is often still problematic in clinical practice: Many geriatricians lack experience in oncology and not all oncologists are familiar with the very specific demands of elderly patients [14, 15]. In addition, under-representation of older cancer patients in randomised controlled trials (RCT), mainly because of pre-existing comorbidities [16], leaves physicians with limited evidence-based recommendations for treating this group of patients. Besides, a comprehensive assessment as described above is quite often not carried out in the clinical practice because advanced age is a priori considered a surrogate marker for functional decline, and hence, such patients are estimated as ‘too impaired’ to tolerate more aggressive therapies. As a consequence of this ‘aegism’, elderly cancer patients frequently do not receive adequate therapy; at the worst, potentially life-saving interventions such as antineoplastic therapy might be withheld. Finally, some physicians seem to think that older patients are no longer interested in potentially straining therapeutic regimens and, therefore, hardly discuss this point with their patients. In fact, with the prospect of life prolongation, the majority of elderly patients were shown to be very willing to undergo such therapies [17]. Taking all these aspects into account, it becomes obvious that deciding on an adequate cancer therapy for elderly patients is a rather challenging process, both for the treating physician and the elderly patient.

## Practical approach for therapy decision in elderly cancer patients, which is constructive for the development of algorithms in elderly MDS patients

**Fig. 1:**
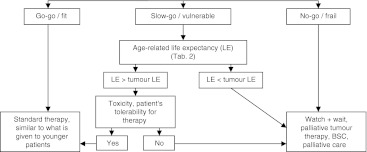
Decision tree for individualised therapy of elderly patients [3]. Based on MGA, three subgroups of patients can be identified. In the final therapy decision, the expectations and wishes of the patient and his/her family are integrated. *BSC* best supportive care, *LE* life expectancy

To support physicians in this balancing act between prescribing the adequate amount of chemotherapy to relieve patients of their cancer symptoms and at the same time not to cause too much harm by toxicity, the following algorithm has been proposed for the clinical practice (Fig. 1) (for review, see [3, 5, 18]. Based on MGA, three groups of elderly cancer patients differing in treatment tolerance should be distinguished (Table 1). The first group comprises the so-called ‘go-go’ or ‘fit’ patients. They are functionally independent in terms of ADLs and IADLs and without serious comorbidities or geriatric syndromes. Group 2 patients (‘slow go’, ‘intermediate’ or ‘vulnerable’ patients) may be dependent in one or more IADLs but not ADLs, and suffer from one to two comorbidities but no geriatric syndromes. Group 3 patients represent the ‘no-go’ or ‘frail’ patients. Mostly, they are ≥85 years, suffer from three or more comorbidities and geriatric syndromes, and are constantly limited in their daily life.

**Table 1:** Classification of elderly cancer patients according to multidimensional geriatric assessment (MGA) (modified from [3])

Category	Parameter	Therapy
Go-go/fit	No functional dependence in ADL (100)	Standard therapy similar to younger patients
	No functional dependence in IADL (8)	
	No relevant comorbidities	
	No geriatric syndromes^a^	
Slow-go/vulnerable	No functional dependence in ADL (100)	Attenuated, individualised therapy
	Dependence in one or more IADL (<8)	
	Comorbidity present but not life threatening	
	Mild memory disorder and depression	
	No geriatric syndromes^a^	
No-go/frail	Age ≥85 years^b^	BSC, palliative care, mild, symptom-oriented therapy
	≥3 grade 3 comorbidities (CIRS-G) or ≥3 comorbidities in Charlson score or one severe comorbidity with constant limitation in daily life	
	One or more geriatric syndromes^a^	

*ADL* activities of daily living, *IADL* instrumental activities of daily living, *CIRS-G* cumulative illness rating scale for geriatrics, *BSC* best supportive care

^a^Geriatric syndromes: dementia, delirium, depression, failure to thrive, neglect or abuse, osteoporosis, falls and incontinence [9]

^b^A higher upper age limit might be considered

Based on this classification, go-go patients should receive full-dose treatment comparable to what is given to younger adults aiming at life prolongation (Fig. 1, go-go/fit) [3, 5, 18]. Before deciding on a therapy for elderly slow-go patients, the age-related life expectancy, as indicated by public age statistics (Table 2), should be compared to the cancer-related life expectancy. If the average age-related life expectancy is above that of the cancer-related, the patient’s treatment tolerance should be evaluated. Depending on this and considering the expectations and wishes of the patient and his/her family, patients should be subjected either to life prolonging or palliative therapies (Fig. 1, slow-go/vulnerable). With a life expectancy below that of the cancer-related, palliation is the therapy of choice. Patients in the no-go group can no longer tolerate a demanding therapy; in most cases, merely palliation comes into question (Fig. 1, no-go/frail) [3, 5, 18].

**Table 2:** Age-related life expectancy in Austria (Statistic Austria 2009; http://www.statistik.at/)

	Remaining years of life at indicated age	
Age (years)	Female	Male
0	82.86	77.42
60	25.09	21.23
70	16.63	13.88
80	9.16	7.68
85	6.2	5.4
90	4.18	3.68

## Challenges in therapy decision for elderly MDS patients

The aforementioned challenges in therapy decision for elderly cancer patients apply in all respects also to elderly MDS patients [19–21]. With this haematologic disease, however, the situation is even worse: While the number of RCTs in elderly cancer patients is low, it is even lower for elderly MDS patients—simply because MDS is a rare disease. In addition, because of this, experience in the therapy of such patients is limited. What makes risk assessment even more complex is the vast number of MDS subtypes in terms of origin, cytological and cytogenetic abnormalities, course of the disease and symptoms. Addressing the latter, MDS classifications available are the French–American–British [22] and the WHO classification [23]. While those two classifications mainly serve for subtyping and have only limited prognostic relevance, the IPSS [24] and the WHO Prognostic Scoring System (WPSS) [25] are risk scoring tools. Of these two, IPSS represents the gold standard for risk assessment of MDS. All these classifications are based on parameters reflecting the biology of the disease, such as bone marrow blasts, chromosomal or molecular aberrations or the consequences thereof, the transfusion requirement or cytopenia. None of these scoring systems, however, considers the special needs and conditions of elderly patients. In addition, recent investigations revealed a considerable impact of comorbidities on the prognosis of elderly MDS patients [9, 10, 26]; these, however, are also not taken into account in conventional MDS assessment tools. Therefore, no specific risk assessment tool for elderly MDS patients exists, although a personalised approach is of particular importance in this group of patients [20, 21].

The author of the current article approached this crucial issue by developing a therapy decision algorithm specifically tailored to the elderly MDS patient that combines MDS classification based on the above-mentioned scores with MGA for individualised risk assessment. Thus, clinical parameters used for the classification of MDS are complemented with individual patient- and age-related factors allowing the development of highly personalised therapies. In this article, this procedure will be described in detail. As decision making is most complex and has more impact on outcome in high-risk MDS patients, the current report focusses on these patients. To illustrate the implementation in the clinical practice, one exemplary case of an elderly female MDS patient, including her geriatric risk level according to this new assessment algorithm as well as her current therapies and state of health, will be presented and discussed in detail.

## New optimised algorithm for personalised decision making in elderly high-risk MDS patients

In a first step, diagnosis and subtype of MDS are defined according to the WHO classification [19, 23]. Secondly, the prognostic score IPSS is applied; by incorporating the transfusion need, the WPSS adds additional information [25]. Risk groups of IPSS comprise low, intermediate-1, intermediate-2, and high-risk patients [24]. In the current approach, the latter two scores (intermediate-2 and high-risk) are merged to a MDS high-risk group. Then, the decision tree for treatment of elderly cancer patients as presented in Fig. 1 is applied. Based on scoring by MGA [5], MDS high-risk patients are classified as go-go, slow-go or no-go (Table 1).

In this context, it should be noted that a clear distinction between MDS- and age-related restrictions is not always possible. An accurate case history, especially regarding the course of the disease, can be helpful. In any case, an attempt should be made to treat MDS-associated complications like, e.g., an infection. Actually, it should be waited with the analysis of the geriatric assessment until the patient has recovered from an acute problem like pneumonia with dehydration to possibly achieve an improvement in the general state of health and then to carry out a new assessment of the overall situation of the elderly patient.

In slow-go patients, age-related life expectancy is evaluated and compared to MDS-related life expectancy as assessed by IPSS. When age-related life expectancy exceeds that of MDS-related life expectancy, treatment tolerance should be assessed taking into account the potential toxicity of the therapeutic regimen as well as preferences and expectations of the patients and their families. With good patient tolerance, treatment regimens can be similar to those suitable for go-go patients; with weak tolerance, type and intensity of therapies should be comparable to those given to no-go patients or to patients with an age-related life expectancy below their tumour-related life expectancy.

## Treatment recommendations for elderly high-risk MDS patients

Specific treatment recommendations for elderly go-go, slow-go and no-go high-risk MDS patients are given in Table 3. Best supportive care (BSC) including red blood cell and thrombocyte transfusions, prophylaxis and treatment with anti-infectives, the interventional use of granulocyte-colonies stimulating factors in neutropenic infections as well as effective iron chelation in case of iron overload (reviewed in 19) represents the basis of effective therapy in any elderly high-risk MDS patient. Although guidelines for effective management of iron overload in MDS patients are available [27], a recent survey among 338 European physicians revealed that half of them are reluctant to initiate iron chelation in MDS patients aged ≥85 years [28].

**Table 3:** Individualised therapy decision in elderly patients (≥70 years) with high-risk myelodysplastic syndromes (IPSS Int-2 and High)

Category	Therapy recommendation	Therapeutic target
Go-go/fit	Best supportive care^a^	Haematologic improvement
	Allo-HSCT^b^	Curation, prolonged OS and PFS
	Azanucleosides^c^	Prolonged OS and PFS, haematologic improvement, relief of symptoms, improved QOL
	Investigational agents^d,e^	Therapeutic target according to aim of the investigational study
Slow-go/vulnerable	Best supportive care^a^	Haematologic improvement
	Azanucleosides^d^	Prolonged OS and PFS, haematologic improvement, relief of symptoms, improved QOL, curation
	Investigational agents^d,e^	Therapeutic target according to aim of the investigational study
No-go/frail	Best supportive care^a^	Haematologic improvement, QOL
	(Azanucleosides)^f^	Improved QOL, haematologic improvement, relief of symptoms
	Investigational agents^d,e^	Therapeutic target according to aim of the investigational study

*Allo-HSCT* allogeneic haematologic stem cell transplantation, *OS* overall survival, *PFS* progression-free survival, *QOL* quality of life

^a^Supportive care represents the basis of all therapeutic options in the distinct treatment arms. Median survival in patients >70 years treated with BSC is 14.4 months in IPSS Int-2 and 4.8 months in IPSS high [24]

^b^Might be feasible in a minority of selected cases with an excellent health status. In these patients an OS at 2-year of >40 % can be achieved in persons aged 65+ [33]. On the relevance of induction chemotherapy prior to HSCT, there is no consensus yet. Hence, decision should be made on an individual basis, possibly after pretreatment with azanucleosides [30, 31]

^c^Azanucleosides such as 5-azacytidine (AZA) (Vidaza®) and 5-azadeoxycytidine/decitabine (DAC) (Dacogen®) are demethylating agents. They have been applied in clinical studies on MDS patients. Vidaza® is approved in this indication in Europe [37], Dacogen® in the USA [39]. The median survival in AZA/BSC only/low-dose Ara-C in the AZA-001 study was 24.5/11.5/15.3 months, respectively [41]; in a subgroup analysis of elderly patients (≥75 years), median OS was not reached at >17 months [41]. Median OS in the French patient-named program was 12.7 months in >70-year-old patients [44]. A prognostic score for patients receiving AZA was developed by Itzykson et al. [44]: three risk groups with OS >24, 15 and 6.1 months, respectively, were defined. Median survival for decitabine-treated patients in the phase-III study by Lübbert et al. [47] was 10.1 months

^d^The inclusion in clinical studies is recommended

^e^Investigational agents include an oral formulation of azacitidine, histone deacetylase inhibitors, lenalidomide and combinations thereof

^f^Even a minor portion of no-go patients might benefit from azacitidine

Therapy of go-go patients should primarily aim to cure the disease. If this is not possible, therapy goals should focus on prolonging overall and progression-free survival (OS and PFS) as well as relief of symptoms and improvement of quality of life (QOL). In slow-go patients, treatment targets are comparable; the curative therapy approach, however, might be realistic only in a minority of cases. For no-go patients, the curative treatment option is not applicable because these patients would not tolerate such demanding therapies. Here, therapeutic targets can only be haematologic recovery, improved QOL and relief of symptoms by supportive care.

Currently, the only potentially curative therapy for high-risk MDS patients is allogeneic haematologic stem cell transplantation (allo-HSCT) [29]. On the relevance of induction high-dose chemotherapy prior to HSCT, there is still no consensus. Preliminary data, however, indicate that azanucleosides prior to allo-HSCT might be a successful option [30, 31]. Decisions in this regard have to be made on an individual basis. In any case, allo-HSCT offers a chance for improved survival with an adequate QOL [32].

Based on their relatively good general condition, only go-go patients will be able to tolerate this procedure (Table 3)—however, only to a minor percentage. Recent extensive analyses revealed no age group differences in reduced-intensity-conditioning HSCT patients [33, 34]. An excellent 2-year survival rate of >40 % was achieved even in patients older than 65 years. Whereas no significant impact of age on non-relapse mortality or OS was observed, the performance status was a major prognostic parameter in this study [33]. Reduced performance status was also an adverse factor in the study carried out by Deschler at al. [32]. In addition to performance status, comorbidities play an important role in the prognosis of elderly MDS patients. Therefore, in addition to age and leukaemia subtype, the HCT-comorbidity index (HCT-CI) was incorporated as a major parameter in a prognosis score [35]. These data support the active consideration of reduced-intensity conditioning HSCT in elderly MDS patients from the go-go group, with comorbidities and performance status playing a more significant role than chronological age. These parameters should be integrated in pre-transplant risk scoring systems. In the future, it will also be important to develop HSCT regimens with reduced intensity while maintaining good efficacy.

In those go-go patients not eligible for allo-HSCT (for whatever reason) and in slow-go patients, azanucleosides, which are supposed to exert their efficacy by DNA hypomethylation, should be considered for therapy [36]. So far, the two agents, 5-azacitidine (AZA) [37, 38] and 5-azadeoxycytidine/decitabine (DAC) [39], have been analysed in MDS patients in clinic studies. AZA, a pyrimidine nucleoside analogue of cytidine, possesses antineoplastic activity (DNA hypomethylation) and exerts cytotoxic effects on abnormal haematopoietic cells in the bone marrow. There is currently no randomised comparison between azanucleosides and HSCT. Studies such as that presently conducted by Kröger will help address this important question (phase II study: 5-azacytidine treatment versus 5-azacytidine followed by HSCT in elderly patients with MDS; www.clinicaltrials.gov, NCT01404741).

With AZA, OS in high-risk MDS patients (int-2 and high-risk according to IPSS) of any age [40], in elderly patients (≥75 years) [41] and in elderly patients (median age, 70 years) with AML according to WHO definition (i.e., AML with 21–30 % BM blasts) [42] was significantly increased when compared to conventional care regimens (CCR) including BSC only, low-dose cytarabine or anthracycline plus cytarabine-based intensive chemotherapy. Subgroup analyses confirmed this survival benefit with AZA also in patients with the poorest prognosis as based on unfavourable cytogenetic profile (−7/del 7q) [40, 43]. Moreover, median time to AML transformation, rates of complete remission, partial remission and any haematologic improvement were significantly higher with AZA than with CCR [40].

In the above-mentioned clinical studies, patients generally had a good Eastern Cooperative Oncology Group (ECOG) performance status (ECOG 0-2). A recent registry on intermediate-2 and high-risk MDS patients receiving AZA included 20 % of patients with a reduced ECOG performance status ≥2 [44], thus reflecting the condition of MDS patients seen in daily practice. In this cohort, a poor ECOG was associated with a poorer OS and a slightly reduced AZA-response in univariate analysis. ECOG status, however, was not an independent prognostic factor in multivariate analyses, and the duration of response was not correlated with an advanced ECOG status. These data point out that patient-related factor such as performance are important determinants of clinical outcome. It has to be mentioned that, in this study, 28 % of the patients received an attenuated AZA schedule due to logistic reasons, older age or renal failure [44]. Hence, it remains unsettled whether elderly patients showed a shorter OS just because of the attenuated AZA schedule or whether the AZA reduction due to advanced age led, in turn, to the reduced OS of these patients. These data as well as a subgroup analysis of this data set regarding patients ≥80 years of age yielding significant overall response rates and OS rates with AZA therapy [45] suggest that an AZA therapy is effective even in elderly displaying a compromised performance status. In addition, an update on this study including also younger patients revealed that advanced age (≤ 70 versus >70 years of age) had no negative impact on response achievement, response duration and OS [44].

Taking all these data together, AZA proved to be the only therapy that significantly prolongs survival in higher-risk MDS patients (int-2 and high-risk according to IPSS) that are not eligible for allo-HSCT. Finally, very recently, a number of prognostic factors for the outcome of an AZA therapy in MDS patients have been identified [44]. Based on these predictive parameters, three risk groups with median OS of >24, 15 or 6.1 months were defined. It is noteworthy that some no-go patients might benefit from AZA (Table 3). For example, when comorbidities are the reason for classification into the no-go group and these comorbidities do not interfere with administration of AZA, improvement of haematologic condition, achievement of transfusion independence and improvement of QOL as well as relief of symptoms can be achieved with this treatment option [40, 46–48].

Even in elderly high-risk MDS patients (≥75 years), the safety and tolerability of AZA were good when compared with CCR, although in the CCR group 67 % of the patients had received BSC only [41]. The most common adverse events of AZA included haematologic (e.g. cytopenias) and non-haematologic events (e.g. gastrointestinal disorders, injection-site reactions) [37, 38]. Most of these adverse events, however, were transient, occurred during early treatment cycles and started to abate already during subsequent treatment cycles [49]. According to its good efficacy and safety, AZA is approved by the European Medicines Agency (EMEA) for intermediate-2 and high-risk MDS patients according to IPSS that cannot receive HSCT [37, 38] and is suggested by actual guidelines also for the use in elderly high-risk MDS patients [1, 46, 50].

The efficacy of DAC in high-risk MDS patients was demonstrated in several clinical studies, including two phase III studies. Remarkably, activity was also demonstrated in patients with unfavourable cytogenetics. An advantage of DAC for elderly patients is also its favourable side-effect profile. However, so far, there has been no clear benefit in terms of survival [51–54]. In a phase III study in elderly patients (median age, 70; range, 60–90 years) completed recently, application of low-dose DAC resulted in improvements in PFS and AML transformation, whereas OS (median, 10.1 months) was non-significantly improved. Remarkably, the QOL was influenced by DAC application, whereby especially the self-reported fatigue and physical functioning were improved. DAC has so far only been approved by the Food and Drug Administration [39] but not yet by the EMEA. The final dose and application schedule of DAC have not yet been conclusively defined and offer scope for future improvements.

Future perspectives regarding further improvements in the therapy of elderly MDS patients might involve oral drugs due to ease of administration. Just recently, promising data from a phase I study on oral AZA have been presented demonstrating bioavailability as well as biological and clinical activity in MDS patients [55]. In addition, combination strategies of AZA or DAC with histone deacetylase inhibitors like valproic acid or vorinostat are in early clinical trials (reviewed in [29]). In high-risk MDS patients with deletion 5q−, lenalidomide seems to be effective [56]. Furthermore, new agents for high-risk MDS patients in clinical trials include the nucleoside analogues clofarabine and sapacitabin as well as tyrosine-kinase inhibitors. Intravenous clofarabine is currently approved for acute lymphoblastic leukaemia in children [57, 58]. While experience is mainly based on therapy of AML, several studies indicate an efficacy also in high-risk MDS patients [59]. Moreover, an oral formulation of clofarabine has entered clinical studies, yielding response rates of 43 % in patients with higher-risk MDS in preliminary analyses [60]. Although response is reduced in patients after administration of DNA methyltransferase inhibitors [61], clofarabine seems to represent a promising drug that exerts activity also in elderly high-risk patients. The optimal dose, administration schedule, combination partners and the appropriate patient population for this therapy, however, remain to be further defined [60, 61]. For the other nucleoside analogue, sapacitabine, currently only very preliminary data are available on its use in MDS and AML patients. Tolerability was good when given orally to patients with refractory–relapse acute leukaemia and MDS [62], and the combination with decitabine was shown to be safe and active in elderly patients with a newly diagnosed AML [63].

In general, however, the number of RCTs on elderly patient is still too low to yield sufficient support for the clinical practice. Although hardly any MDS study defines an upper age limit, elderly patients are often not enrolled because of multiple comorbidities [16]. The typical MDS patient in clinical practice, however, exhibits exactly these two characteristics: advanced age and multiple comorbidities. Hence, participation of unfit, elderly MDS patients in clinical trials should definitely be increased.

## Exemplary case of a go-go, high-risk MDS patient

An 84-year-old lady presented with a transfusion-dependent anaemia. The patient’s bone marrow was characterised by dysplastic features and the presence of 19 % blasts, thus leading to the diagnosis of an MDS (RAEB II) in 01/2009. Based on a normal karyotype and the presence of a granulopenia, an IPSS Intermediate-2 was diagnosed. Her comorbidities were minor, resulting in a Cumulative Illness Rating Scale for Geriatricians (CIRS-G) total score of 3 [CIRS-G categories grade 3/4, 0; Charlson Comorbidity Scale, 0; Hematopoietic Cell Transplantation Comorbidity Index (HCT-CI), 0]. Geriatric assessment revealed excellent functional (WHO performance status, 1; Karnofsky status, 90; ADL, 100/100; IADL, 8/8; timed up and go, 6.8 s) and cognitive capacities (Mini-Mental State Examination, 30/30). Geriatric Depression Scale (GDS) disclosed no evidence for depression (GDS, 1/30). Social support was excellent (Social Support Questionnaire F-Sozu, 4.405). Thus, geriatric assessment as described recently [64, 65] resulted in assignment to the go-go group.

An MDS IPSS Int-2 is associated with a median survival of 1.2 years in patients 70+ years [24]. Based on an age-adjusted life expectancy of 6.2 years for an 84-year-old woman in Austria (Table 2), the MDS diagnosed resulted in an estimated loss of five life years in this patient. Similarly, application of the prognostic model of Naqvi et al. [10] resulted in a significant loss of life years (intermediate category: median OS, 23 months). The clinical situation was comprehensively discussed with the patient. Arguments to start a therapy with AZA were the patient’s low QOL caused by the high transfusion need, her wish to receive a therapy and the good chance of a response based on the predictive score suggested by Itzykson et al. [44]. Thus, a therapy with AZA was started in 05/2009 at a standard dose of 75 mg/m^2^ subcutaneously. The first cycle was applied in a 1–7 schedule; all subsequent cycles on an ambulatory basis in a 5–2–2 scheme. Due to thrombopenia, a dose reduction was performed, resulting in a final dose of 33 % in cycle 6 and all subsequent cycles. Up to now (03/2011), 21 cycles have been administered to the patient. Due to granulopenia, an antimicrobial prophylaxis with itraconazole, valaciclovir and sulfametrol/trimethoprim was performed from initial diagnosis until cycle 10 of the therapy. Subjective side effects of AZA included a minor local reaction at the injection site [Common Terminology Criteria (CTC), grade I]. Antiemetic prophylaxis was performed with intravenous tropisetron resulting in excellent control of chemotherapy-induced nausea and vomiting. Attempts to discontinue tropisetron, however, resulted in CTC grade II vomiting. Grade 3 and 4 adverse events were haematologic. Here, however, it has to be kept in mind that with pancytopenia being the lead symptom of MDS, a treatment-related cytopenia can hardly be distinguished from a disease-related one.

**Fig. 2:**
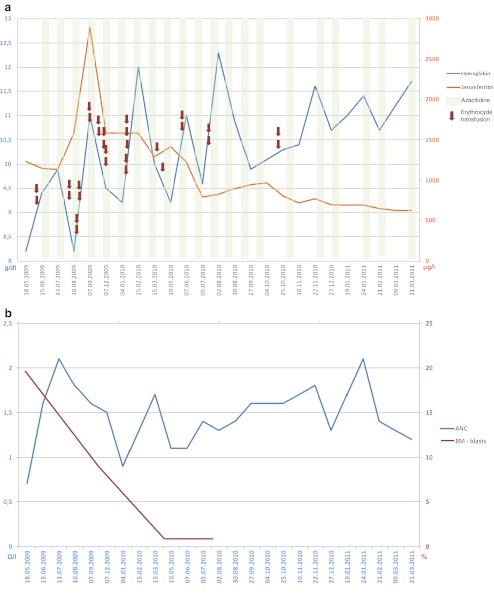
Case presentation of an 84-year female MDS patient, RAEB II, IPSS intermediate-2, treated with AZA. The time course of red blood cell transfusions, of AZA application, the subsequent response in haemoglobin, serum ferritin (**a**) and in ANC and BM blasts (**b**) are demonstrated

AZA therapy was very effective, yielding a marked increase in granulocytes and haemoglobin as well as a pronounced reduction in the transfusion need, thus fulfilling the International Working Group criteria 2006 [66] of a stable disease with haematologic improvement (Fig. 2). Moreover, bone marrow blasts decreased from 19 to 7 to <5 % fulfilling the criteria of a bone marrow complete remission. Due to elevated serum ferritin levels, a successful iron chelation with deferasirox 500 mg/day was started as suggested by the Austrian MDS-Platform [27].

This case highlights that a therapy with AZA is feasible, advisable and effective even in a very old patient suffering from high-risk MDS. Biological age as defined by MGA assessment and predictive parameters for AZA response [44] should form the basis for decision-making. Up to now (03/2011), cytopenias of the patient have improved, and the transfusion requirement has decreased considerably. Compared to the cancer-related life expectancy, life-prolongation of more than 1 year could be achieved. The therapy with AZA is being continued.

## Summary and outlook

How to deal with high-risk MDS in the elderly? The current article approached this question by presenting a new algorithm that will help physicians in clinical practice to identify the best possible therapeutic approaches for this specific group of patients. By directly linking MDS classification with geriatric risk assessment and integrating not only chronological age but also aspects of age-adjusted life expectancy, functional capacities and comorbidities in the geriatric assessment, the specific needs and conditions of each individual patient can be precisely evaluated. Allo-HSCT is the only curative approach for MDS. However, besides comorbidities and a poor performance status, advanced age is one of the major reasons why most of the MDS patients are not eligible for allo-HSCT. Addressing this issue, several alternative therapeutic options for elderly and frail MDS patients are presented here, including the hypomethylating agent azacitidine. Still, there is an urgent need for the implementation of official recommendations and guidelines for the treatment of this specific group of patients. Besides the above-mentioned parameters, such guidelines should also address the impact of comorbidities, particularly renal, cardial and cognitive impairment, as well as existing neuropathy, on the tolerability of elderly patients. For this, clinical studies in elderly and in unfit MDS patients have to be performed as well as basic research to better understand ageing, frailty and their interface with cancer. We have just started to successfully integrate paradigms and concepts of geriatric oncology in the evaluation and treatment of elderly MDS patients. Ongoing close cooperation with geriatricians and the propagation of individualised treatment for all elderly cancer patients will further support these efforts!
